# Molecular Aspects of Colorectal Adenomas: The Interplay among Microenvironment, Oxidative Stress, and Predisposition

**DOI:** 10.1155/2020/1726309

**Published:** 2020-03-16

**Authors:** Gitana Maria Aceto, Teresa Catalano, Maria Cristina Curia

**Affiliations:** ^1^Department of Medical, Oral and Biotechnological Sciences, G. d'Annunzio University of Chieti-Pescara, 66100 Chieti, Italy; ^2^Department of Clinical and Experimental Medicine, University of Messina, 98125 Messina, Italy

## Abstract

The development of colorectal cancer (CRC) is a multistep process initiated by a benign polyp that has the potential to evolve into *in situ* carcinoma through the interactions between environmental and genetic factors. CRC incidence rates are constantly increased for young adult patients presenting an advanced tumor stage. The majority of CRCs arise from colonic adenomas originating from aberrant cell proliferation of colon epithelium. Endoscopic polypectomy represents a tool for early detection and removal of polyps, although the occurrence of cancers after negative colonoscopy shows a significant incidence. It has long been recognized that the aberrant regulation of Wingless/It (Wnt)/*β*-Catenin signaling in the pathogenesis of colorectal cancer is supported by its critical role in the differentiation of stem cells in intestinal crypts and in the maintenance of intestinal homeostasis. For this review, we will focus on the development of adenomatous polyps through the interplay between renewal signaling in the colon epithelium and reactive oxygen species (ROS) production. The current knowledge of molecular pathology allows us to deepen the relationships between oxidative stress and other risk factors as lifestyle, microbiota, and predisposition. We underline that the chronic inflammation and ROS production in the colon epithelium can impair the Wnt/*β*-catenin and/or base excision repair (BER) pathways and predispose to polyp development. In fact, the coexistence of oxidative DNA damage and errors in DNA polymerase can foster C>T transitions in various types of cancer and adenomas, leading to a hypermutated phenotype of tumor cells. Moreover, the function of Adenomatous Polyposis Coli (*APC*) protein in regulating DNA repair is very important as therapeutic implication making DNA damaging chemotherapeutic agents more effective in CRC cells that tend to accumulate mutations. Additional studies will determine whether approaches based on Wnt inhibition would provide long-term therapeutic value in CRC, but it is clear that *APC* disruption plays a central role in driving and maintaining tumorigenesis.

## 1. Introduction

Colorectal cancer (CRC) is the third most common cancer worldwide. In fact, CRC is the third most commonly diagnosed cancer in males and the second in females, with 1.8 million new cases and almost 861,000 deaths in 2018 according to the World Health Organization [1]. The risk of developing CRC is influenced by environmental and genetic factors. The incidence and mortality rates of CRC vary widely worldwide. Current literature suggests that incidence rates are constantly increased for patients diagnosed under the age of 50, who often present with symptomatic diagnosis and with a more advanced tumor stage [2]. Up to 35% of CRCs are thought to be due to heritable factors, but currently, only 5% to 10% of CRCs are attributable to high-risk mutations in known CRC susceptibility genes.

The development of CRC is considered to be a multistep process, initiated by the development of a benign polyp that has the potential to evolve into an *in situ* carcinoma by the accumulation of additional somatic mutations [3]. The majority of CRCs arise from colonic adenomas, originating from aberrant cell proliferation of colon epithelium.

Adenoma prevalence is about 40% in Western populations, ranging from 50 to 75 years, and is prevalently male associated [4]. Screening and surveillance programs are useful to identify precursor lesions and to prevent the death from CRC. Endoscopic polypectomy represents a tool for early detection and removal of polyps. The occurrence of cancers after negative colonoscopy shows a significant incidence since adenomas could be missed during colonoscopy or biological changes could occur in tumor growth rates [5].

Many risk factors for polyp development have been correlated to lifestyles and can play a potential role in gut mucosal inflammation consequent to dysbiosis [6, 7]. Altered microbiome composition is associated with signal activation involving mitochondria, or/and altered redox homeostasis, proinflammatory cytokines induction, and stimulation of the immune system [8]. It is well known that chronic inflammation and reactive oxygen species (ROS) production in the colon epithelium can impair the Wingless/It (Wnt)/*β*-catenin [9] and/or base excision repair (BER) pathways [10, 11], enhance a cascade of molecular reactions in cells, and alter the metabolic state of tissues and predispose to polyp development.

Adenomas represent the main precursors of CRC in high-risk family groups with a history of polyposis syndrome and in the wide-ranging population. Indeed, genetic events like gain or loss of functions on molecules, necessary for functional homeostasis of the intestinal cells, can lead to the polyp development [12].

For this review, we will focus on the development of adenomatous polyps through oxidative stress and signaling in the colon epithelium interactions. The current knowledge of molecular pathology will be highlighted with particular regard to the relationships between oxidative stress and other risk factors, like lifestyle, microbiota, and predisposition. In fact, only the understanding of the complex interrelations between these factors and the body's response and immune defense will allow correct prevention of early events in colon carcinogenesis.

## 2. Epidemiology and Risk Factors of Intestinal Polyps

Although the development of polyps is strongly correlated with the development of CRC, their malignant potential differs among different subtypes [13].

At least three subtypes of polyps can be distinguished on the basis of histology and the underlying molecular pathway: tubular/villous adenomas, hyperplastic polyps, and sessile/traditional serrated adenomas. Tubular/villous adenomas are characterized by an adenomatous histotype, whereas both hyperplastic polyps and sessile/traditional serrated adenomas have a serrated histotype [14]. The prevalence of hyperplastic polyps is higher than that of tubular/villous adenomas and sessile/traditional serrated adenomas [13]. Although an increased risk for malignant transformation has been described for hyperplastic polyps, their tumorigenic potential is considered to be lower than that of tubular/villous adenomas and sessile/traditional serrated adenomas [13]. In addition, the risk of developing cancer is strongly associated with the number and size of previously encountered polyps [15]. Therefore, the development of multiple colonic polyps with malignant potential will result in an increased lifetime risk of developing CRC. Owing to the malignant potential of tubular/villous adenomas, patients diagnosed with adenomatous polyposis, i.e., the constitutive development of multiple colorectal adenomas, are at increased risk of developing CRC.

The different histological types of polyps show a variable anatomic distribution in the large bowel. Even though the different types of polyps may be disseminated in all the large bowel, adenomas and hyperplastic polyps are prevalently located in distal colon [16–18], and sessile serrated polyps are often found in proximal colon and they seem to be the precursor of up to 30% of CRC [19–23]. In a study conducted in an Italian population, serrated lesions were recognized in the proximal colon in 38% of cases, while 40.7% were in the left colon, and 14.3% in the rectum [24]. Various risk factors, unmodifiable and/or modifiable, may influence polyp onset. The unmodifiable factors are represented by age, gender, and ethnicity while the modifiable ones, including unhealthy diet, tobacco smoking, excessive alcohol consumption, physical inactivity, and obesity, have been independently associated with the risk of polyps and altered composition of the intestinal microbiota [25] (Table 1).

### 2.1. Unmodifiable Factors

The prevalence rate of polyps increases with age, and their major incidence occurs after 50 years [28], with a peak at 70 years [16]. Colorectal polyps are uncommon before age 40, but some reports indicate a rising incidence of them before 50 years [56, 57] and even among young patients from 20 to 39 years of age, with a prevalent detection of serrated lesions [26, 27]. Unlike most studies that affirm that adenomas are more common in males than in females [16, 29], Klein et al. highlight the prevalence of adenomas in the left colon in women compared with men [18], whereas Brettahuer et al. did not observe any significant sex differences [30]. In contrast, sessile serrated polyps were commonly detected in females in the proximal colon [22]. Several studies demonstrated a significant association between ethnicity and risk to develop polyps. The prevalence of colorectal polyps may vary across countries and racial/ethnic groups. High incidence of colorectal polyps is found in Western countries and ranges from about 30% to 50% of the population [4, 30]. Solakoğlu et al. identified a higher percentage (81%) of adenomas in patients through a screening colonoscopy based retrospective study conducted in Turkey [57]. African Americans show a higher incidence of more aggressive colorectal polyps than Caucasians and prevalently located in the proximal colon [58]. In particular, blacks have an increased prevalence of precursor lesions >9 mm as compared to white individuals [31] and show a higher number of polyps at a young age compared to Hispanic Americans [59], with whom they share an increased risk of adenomas than whites at an older age [60]. Katsidzira et al. conducted a study that demonstrated racial differences in the incidence of polyps in Zimbabwe: a less occurrence of polyps among black Africans (5%) and more frequent onset of adenomatous polyps in Caucasians (8%) and Asians (9%), whereas colorectal cancer was more diffused among black Africans [61]. These geographic differences might be supported by diversities in lifestyles and exposition to different environmental factors. Indeed, there is a large body of evidence that correlates modifiable aspects as dietary habits, or less modifiable aspects as pollutant exposure, with modifications of the structure of the gut microbiota, inducing carcinogenesis of the colorectum.

### 2.2. Environmental Factors

Environmental influences alone or in association with lifestyle may be responsible for the development of colorectal polyps. An increased risk of polyps in the large intestine is associated with chronic low-dose exposure to persistent organic pollutants, such as organochlorine pesticides (OCPs) and polychlorinated biphenyls (PCBs). Lee et al. reported a strong association of colorectal polyps and cancer with the OCP *β*-hexachlorocyclohexane, as well as low-chlorinated PCBs [48]. The OCP *p*, *p*′*-*dichlorodiphenyldichloroethylene (*p*,*p*′-DDE), considered the main metabolite of dichlorodiphenyltrichloroethane (DDT), could induce the activation of Wnt/*β*-catenin and Hedgehog/Gli1 (Hh) pathways, resulting in the overexpression of c-Myc and cyclin D1 and adenocarcinoma cells proliferation. *p*,*p*′-DDE increases ROS levels in colorectal cancer cells by NADPH oxidase (NOX) activation and reduces the expression of antioxidant enzymes [32]. PCBs are also considered potent activators of oxidative stress by increasing the activity of NOX through phosphorylation of p47^phox^ [62].

PCBs are responsible for altered gut barrier functions and permeability by modifying the expression of tight junction proteins in the intestinal mucosa [62]. These evidences suggest that a similar mechanism could predispose to the gut inflammation state and formation of colorectal polyps. Moreover, xenobiotics, as persistent organic pollutants, can interact with gut microbiota modifying its structure and inducing carcinogenesis of colorectum [63]. OCPs are absorbed in the small intestine and accumulate mainly in the adipose tissue, from which they join the bloodstream [64], whereas PCBs are mainly removed from the body in feces [48]. Dioxin-like PBS126 exposure significantly alters the gut microbiota equilibrium, predisposing to intestinal inflammation [33].

Heavy metal contact consequent to food uptake or occupational exposure is considered responsible for gut microbiota dysbiosis. Metabolites of arsenic alter gut bacterial species, the production of butyrate, and the gut-associated immune system [34]. Cadmium exposure determines an inflammatory response and tight junction alterations in the intestinal barrier that increase gut permeability and favour the *Bacteroidetes* in the microbiome [35]. Antibiotics, nanoparticles as environmental pollution, and additives used for food preservation influence and alter the composition and function of the gut microbiota, predisposing to the onset of diseases [36].

### 2.3. Modifiable Factors

In recent decades, extensive epidemiological and experimental researches have highlighted the key role of the diet in preventing the CRC onset. Dietary behaviours influence the composition of gut microbiota. Indeed, bacterial metabolism of bile acids, heme iron, and complex carbohydrates is crucial for barrier function and immune homeostasis and inflammation. The illness has been ascribed to the excessive consumption of red and processed meat [65], which is associated with an elevated risk of adenomas in the descending and sigmoid colon [37]. Voskuil et al. in a case-control study suggest an association between habitual methods of meat preparation and adenoma formation [66]. In fact, the daily intake of red meat cooked at high temperature, with production of polycyclic aromatic hydrocarbons such as benzo(a)pyrene, or saturated fat consumption, low fiber diet [67, 68], heterocyclic amines produced during cooking of red meat [38], and N-nitroso compounds can induce genotoxic effects [69]. In particular, the risk for single adenomas was found to be associated with the heterocyclic amines 2-amino-3,8-dimethylimidazo[4,5]quinoxaline (MeIQx) and 2-amino-1-methyl-6-phenylimidazo[4,5-*b*]pyridine (PhIP) and benzo[a]pyrene [37]. The exposure to benzo[a]pyrene generates intestinal inflammation and also gut microbiota modifications [39]. Heterocyclic amines introduced by diet are metabolized and converted to mutagens and DNA adducts by microbe intestinal flora. Indeed, diet prevalently based on red meat and animal fats promotes dysbiosis with a selection of bacterial species that alter bile acid metabolism, with proinflammatory and prooncogenic effects [40]. A crucial role in the colorectal adenoma risk is also referred to a diet rich in heme iron contained in red meat that leads to modification gut barrier homeostasis and microbiota associated with the selection of bacteria linked to inflammation and colorectal adenoma [41]. Heme iron induces the oxidation of dietary polyunsaturated fatty acids and the consequent production of unsaturated aldehydes, such as malondialdehyde and 4-hydroxynonenal (HNE), which are cytotoxic [42]. Colon cells harbouring Adenomatous Polyposis Coli (*APC*) mutations in rats are resistant to apoptosis induced by HNE, suggesting that lipoperoxidation by heme iron confers to these cells a selective survival advantage [70]. Luminal lipoperoxidation is associated with high levels of mucosal inflammation markers, such as the myeloperoxidase activity with ROS production, increased gene expression of interleukin 6 (IL-6) and transforming growth factor *β* (TGF-*β*), and enhanced cellular permeability due to reduced expression of the junctional adhesion molecules [41].

The toxicity of the nitrosylated heme iron seems to act for site-specific etiology of colon adenoma. In fact, an increased risk is related to nonnitrosylated heme iron uptake with advanced distal adenoma, as well as nitrosylated heme iron with proximal adenoma [42].

Resistant starch stimulates gut bacterial fermentation to produce short-chain fatty acids (SCFAs) into the colon, which are known to modulate immune responses in the intestine [71]. SCFAs act as a gut barrier by reduction of oxygen concentrations and induction of hypoxia-induced factor (HIF). Nevertheless, butyrate one of SCFAs shows a “paradox” behaviour since it prevents intestinal polyp formation or modulates the expression of genes to elude the aberrant cell colon proliferation through the activation of apoptosis or upregulation of detoxifying enzymes [72]. Botma et al. observed an increased risk of colorectal adenomas associated with a “snack” dietary pattern in a prospective cohort study involving patients affected with Lynch syndrome [73]. High-fat diet intake can increase the expression of macrophage markers and inflammatory mediators in the adipose tissue, which are associated with an increased number of large polyps [43]. Moreover, increased consumption of fat is associated with high production of hydrogen sulphide, inducing inflammation and cell proliferation, by sulphate-reducing bacteria [51, 54].

Another risk factor for colorectal adenoma development is the lack of physical activity [44]. Several biological mechanisms have been proposed to explain the impact and the protective effect of the physical activity on the less likely development of colorectal adenomas. Active lifestyle confers health benefits since it increases the intestinal motility and the immune system functions, decreases the systemic inflammation, reduces the insulin resistance and the obesity, and increases the activity of free radical scavenger in the antioxidative systems [74, 75].

A study highlighted the association of total stressful life events and colon polyps in African Americans, supporting the evidence that stress hormones alter the rate of cell growth and proliferation [45]. A study based on a multiethnic colorectal screening [46] observed that individuals with an active lifestyle showed a significantly lower prevalence of adenoma risk, compared to counterparts who had reduced physical activity and demonstrated the development of adenomas in distal colon prevalently. Consistent with these findings, weight loss associated with physical exercise has been found to reduce biomarkers of oxidative stress, such as oxidized low-density protein (LDL), in postmenopausal women [76]. Significant inverse association of physical activity and colon adenoma suggests the importance of its role in colon cancer prevention, particularly in males [77]. During adolescence, physical activity can reduce the risk of colorectal adenoma later in life [78].

In obese subjects, the increased body mass index ≥30 (BMI) or overweight (25 ≤ BMI ≤ 29.9) is considered a risk factor for colonic adenomas [79]. Botma et al. found a significant association between BMI and increased risk of adenomas in men, in a prospective cohort study conducted on carriers of DNA mismatch repair (MMR) gene mutations [80].

A recent case-control study involving African Americans showed an increased risk of colorectal adenoma onset associated with high circulating levels of TNF-*α*, IGF-1, and the metabolic biomarker adiponectin, which is secreted from the abdominal fat tissue and can induce cell proliferation [47]. Numerous studies analyzed the relationship among the metabolic syndrome associated-diseases, such as obesity, hypertension, and diabetes mellitus, and the development of colorectal adenomas, as well as the association of high triglyceride/high-density lipoprotein cholesterol ratio with serrated polyps [81, 82].

In recent years, there has been a growing interest in the understanding of the relationships between the hepatic manifestation of the metabolic syndrome, represented by nonalcoholic fatty liver disease (NAFLD), and the development of colorectal polyps [83, 84]. NAFLD is a significant risk factor for adenomatous and hyperplastic polyps in males as compared with females [85, 86], although Li et al. had demonstrated that NAFLD is a greater factor for the adenomatous polyp development in women than in men [87]. Moreover, an association among NAFLD, alcohol consumption, and colorectal adenomas has been identified [88] but has not still been well investigated. Randomized controlled studies on the interactions among physical activities, dietary factors, modulation of immune responses, and the microbiota will be crucial to advance the customized guidelines in the prevention of colorectal adenomas.

Smoking and alcohol consumption increased the risk of adenomas. The association between smoking and adenomas has been pointed out in a prospective cohort study [49]. Cigarette smoking components, such as benzo[a]pyrene and heterocyclic amines, represent a significant modifiable risk factor for polyps since they induce chronic inflammation consequent to oxidative stress and genetic/epigenetic alterations. In particular, tobacco use is associated with the development of serrated polyps [48, 50] and with the development of large and flat colorectal polyps [89]. A study of Fu et al. described a strong association between cigarette smoking and synchronous hyperplastic polyps and adenomas, due to a stronger relationship of cigarette smoking with hyperplastic polyps than with adenomas [25].

Alcohol is metabolized by bacteria expressing alcohol dehydrogenase that converts ethanol to acetaldehyde, which is a carcinogenic factor. Moreover, alcohol interferes with folate metabolism that is involved in DNA methylation [51]. Diergaarde et al. correlated an increased risk of adenomas with alcohol assumption and smoking and also a decreased risk of adenomas with a diet enriched with fruit and fibers in a Dutch case-control study involving individuals affected with hereditary nonpolyposis colorectal cancer (HNPCC) [90]. The association was not significantly confirmed by Winkels et al. [49].

## 3. Gut Microbiota

The colonic mucosa is in contact with the intestinal bacteria and their metabolic products. Normal microbiota is composed of obligate anaerobic bacteria. The gut microbiota plays a pivotal role in physiological homeostasis of the intestine by the modulation of immune responses, enhancement of epithelial barrier function, and stimulation of cell proliferation. Alterations in the gut microbiome (dysbiosis) drive the signals between mitochondria and epithelial mucosal cells and induce inflammasome signaling through the activation of immune cells until they change the epithelial barrier function [8]. In the past few years, several studies have definitively shown that gut microbes exert distinct impacts on DNA damage, DNA methylation, chromatin structure, and noncoding RNA expression in colon epithelial cells [91]. Some genes and pathways that are altered by gut microbes are also related to CRC development, particularly those involved in cell proliferation and Wnt signaling [91]. Dysbiosis is the consequence of diet factors or potentially harmful microorganisms which can induce inflammatory processes. Among them, *Fusobacterium nucleatum* (*Fn*), an intermediate and driver microbe, attaches to the colon epithelial cells by its adhesion molecule FadA that interacts with E-Cadherin, inhibiting its oncosuppressive activity [52]. E-Cadherin is known to maintain the integrity of mucosa at cell-cell junctions of colon epithelial cells, whereas the binding with FadA increases the endothelial permeability and allows activating *β*-catenin and uncontrolled cell proliferation, predisposing the host to the development of adenomas, induces oxidative stress, and stimulates the immune system [51]. Altered functions of the intestinal barrier allow other bacteria, called passengers, to cross the colon epithelium. Indeed, dysregulation of the barrier is associated with the increased production of IL-23 [92] and the induction of proinflammatory cytokines, including IL-17 that generates a proinflammatory microenvironment recruiting tumor-infiltrating immune cells (i.e., tumor-infiltrating lymphocytes, TILs) [6, 7, 51]. In addition, McCoy and coauthors observed a significant positive link between IL-10 and TNF-*α* gene expression and *Fn* in colorectal adenomas, suggesting their potential role in gut mucosal inflammation [93]. Proinflammatory cytokines can also induce DNA methyltransferases (DNMT) with the silencing of tumor suppressor genes. Inflammation associated with *Fn* activates the Wnt/*β*–catenin pathway through the production of chemical mediators as cyclooxygenase-2 (COX-2) and Prostaglandin E2, producing a tumor microenvironment and finally promoting CRC progression. Indeed, COX-2 generates reactive aldehydes that modify proteins, induce damage DNA, and can also activate RAS and PI3K signaling pathways. This scenario is also associated with ROS production by inflammatory cells.

*Escherichia coli* (*Ec*) is a gut commensal and its oncogenic potential is linked with the ability of some strains to produce toxins such as cytolethal distending toxin and colibactin, which promote inflammation and are involved in colon carcinogenesis through DNA breaks and mutations [51, 54]. Colibactin is a genotoxin encoded by the multienzymatic machinery tumor-promoting polyketide synthase (pks) islands that increase cell proliferation and are characterized by double-stranded DNA breaks and impaired DNA repair [53, 54].

Enterotoxigenic *Bacteroides fragilis* (*Bf*) secretes the Bacteroides fragilis toxin (BFT) and is associated with inflammatory Th17 cells. It activates spermine oxidase (SPO) with ROS production and DNA damage [54]. BFT binds to a specific colon cell receptor and activates Wnt and NF-kB signaling pathways. This determines an increase in cell proliferation, the production of proinflammatory mediators, and DNA damage [53]. Among other bacteria, *Enterococcus faecalis* (*Ef)* produces superoxide that induces DNA mutations, while *Helicobacter pylori* (*Hp*) infection is associated with an increased risk of serrated polyps [55].

Signs of dysbiosis occur early in the colorectal adenoma-carcinoma sequence so that the mucosal microbiota shows distinct structural modifications during the different steps of colorectal carcinogenesis [94]. Adenomas were found to be rich in *Escherichia coli*, *Pseudomonas veronii*, and members of the *Enterobacteriaceae*, while *B. fragilis* increases during adenoma-to-carcinoma progression [94].

Differences in gut bacteria composition were detected in patients with various histological types of polyps. *Fn*, Ef, *Streptococcus bovis* (Sb), enterotoxigenic Bf, and lower numbers of *Lactobacillus* spp., *Roseburia* spp., and *Bifidobacterium* spp. were detected in the colon of patients with tubular adenoma and villous/tubulovillous polyps compared to healthy subjects and patients with hyperplastic polyps or sessile serrated polyps [95]. These findings suggest that microbiota affects the development of adenomatous polyps, but not sessile serrated adenomas, which are located in specific areas of colorectum [96].

Microenvironment homeostasis and differential expression of Wnt signaling components are influenced by bacterial colonization [97]. Furthermore, functional interactions were suggested by the association between loss-of-function mutations in tumor pathway genes (including Wnt) and displacements in the abundance of specific sets of bacterial taxa [98]. In the intestinal epithelium, the expression of antimicrobial peptides regulates defense against infection and homeostasis thanks also to the cholinergic nervous system that controls antimicrobial gene expression. The release of acetylcholine from neurons induced by infections can stimulate muscarinic signaling in the epithelium, inducing downstream the expression of the canonical Wnt signal which, by determining the expression of type C lectin and lysozyme, integrates the host defense [99].

In animal models of colon carcinogenesis, the use of nonsteroidal anti-inflammatory drugs (NSAIDs) can inhibit antigen-presenting cells and control intestinal inflammation and intestinal immune homeostasis via the canonical signal Wnt [100]. In fact, commensal-polarized macrophages induce gene mutation, chromosomal instability, and endogenous transformation through microbiome-induced bystander effects (MIBE) that activate Wnt/*β*-catenin signaling [101]. The administration of NSAIDs may contribute to the downregulation of the canonical Wnt/*β*-catenin pathways acting as PPAR agonists, which can promote cell cycle arrest, cell differentiation, and apoptosis and reduce inflammation, oxidative stress, proliferation, invasion, and cell migration [102].

In addition, the type I interferons (IFNs) produced in the gut under the influence of microbiota play an important role in controlling the proliferation and function of the intestinal epithelium in the context of *β*-catenin activation [103]. The mechanism of bacterial production of ROS in phagocytes in response to ligand binding with formyl peptide receptors (FPR) and subsequent activation of NADPH oxidase 2 (Nox2) was well defined while the response to microbial signals from Nox1 has not been fully investigated in epithelial cells [104].

ROS enzymatically generated may modulate many signal transduction pathways inducing transient oxidation of sensitive thiol groups in sensory proteins. Examples of redox-sensitive proteins include tyrosine phosphatases that act as MAPK pathway regulators, focal kinase adhesion, and components involved in NF-kB activation [104].

Changes in gut commensal bacteria environment can confer resistance to/or promote infection by pathogenic bacteria and activate inflammation signaling by toll-like and interleukin-1 receptors (TLR and IL-1R) [105]. This is further evidenced by the observations that tumor suppression activity has been demonstrated by some negative TLR and IL-1R signaling regulators and commensal bacteria in the gastrointestinal tract. Modulators of innate immunity can act as a bridge between the inflammatory signaling TLR/IL-1R and the oncogenic RAS signaling pathway, which represents the first necessary path to the onset of colon cancer [106, 107].

## 4. Interplay between Colon Epithelium Renewing and Oxidative Stress

The colon epithelium is constantly renewed and arises from only a few intestinal stem cells residing at the crypt base. The epithelial cell layer is derived and is progressively differentiated from these amplifying cells until the top of the villi [108]. The stem cell niche is a microenvironment required to maintain the “staminality” of a stem cell proper lineage ratios; it also supports the absorptive, secretory, and barrier functions [109].

The maintenance and regeneration of epithelial organs take place through the simultaneous production of proliferative and differentiation signals. Deregulation of these signals in intestinal epithelium leads to the genesis of small lesions called aberrant crypt foci, whose expansion causes the adenoma that can progress to *in situ* carcinoma and then to invasive adenocarcinoma [110]. Molecular studies have long defined that these stages on the development of colon cancer are driven by a progressive accumulation of somatic/genetic alterations which confers an advantage in uncontrolled growth [111]. Besides the mechanistic studies focused on the intrinsic alterations of tumor cells, in recent years, the involvement of the activation of the local rather than peripheral immune response has been considered as a fundamental component of the prevention of neoplastic transformation of the epithelium of the colon. The theory of immune-surveillance, postulated for several decades ago, hypothesized that a normal cell that acquires oncogenic mutations can be recognized as foreign and eliminated by the immune system [112, 113]. Actually, different findings support this theory, since cancer progression resulted in changes in the composition of tumor-infiltrating cells in the suppressive immune microenvironment [114].

### 4.1. Self-Renewal Dysregulation of the Intestinal Epithelium

The rapid self-renewal of the intestinal epithelium and the differentiation gradient in the crypt-villus structure is controlled by the integration of multiple and redundant cell signaling pathways, such as PI3K/AKT, ERK1/2, *β*–Catenin/GSK3, SMAD, NICD, and JAK/STAT1, which are activated via mechanisms of hormesis by paracrine ligands released from all cells of the tissue community. Finally, the levels of the first and second messengers elicit the effects on the intestinal epithelium renewal and differentiation [115]. In the colon mucosa, the control of the balance between differentiation and renewal is mainly guided by Hedgehog (Hh), bone morphogenetic protein (BMP), Notch, Hippo, and Wnt signaling pathways [108, 116–120].

The Hedgehog signaling acts as a prodifferentiative force in the development and normal homeostasis of the intestinal epithelium. The expression signatures suggest that stromal Hh signaling activity exerts a prodifferentiative influence on intestinal epithelial stem cells, mediated at least in part by the modulation of BMP signaling factors secreted by stromal cells that negatively regulate self-renewal of Lgr5+ intestinal stem cells, and constrains the expansion of intestinal epithelium, therefore attenuating colorectal cancer formation [121]. In the adult intestinal homeostasis, a reduction of Hh signaling increases Wnt activity and intestinal stem cell compartment [122–124].

The Hippo kinase cascade pathway promotes the cytoplasmic localization of YAP/TAZ, which restricts cell proliferation and induces apoptosis. The enterocyte self-renewal and crypt regeneration are triggered by YAP through Wnt/*β*-catenin signaling. This mechanism also stimulates epithelial cell proliferation following epithelial damage and may facilitate the promotion of associated cancer development to colitis through signals of chronic inflammation and excessive tissue regeneration [125].

Differentiation and proliferation of the epithelium in the intestine are redox-sensitive and regulated by NADPH oxidases [126, 127]. These physiological processes must be highly controlled and balanced. The ROS produced by enzymes of the NADPH oxidase family behave as second messengers for cellular signaling [128].

In the gut, NADPH oxidase 1 (NOX1) is the major expressed NADPH oxidase and, together with p47^phox^ (alias Neutrophil cytosol factor 1) and NADPH oxidase organizer 1 (NOXO1), mediates ROS formation that facilitates the proliferation of colon epithelial cells [129]. NOXO1 acts as a mediator of constant redox-dependent signaling in the differentiation, proliferation, or cell survival. In epithelial cells, the absence of NOXO1 promotes proliferation and reduction of apoptosis supporting malignant transformation and tumor development [129]. NOXO1 is also needed to maintain the activity of the Notch signaling pathway by enabling the activity of a disintegrin and metalloproteinases (ADAM) [130].

Notch also plays an important role in the renewal of the colon epithelium. It is conserved in the cell-cell communication pathway and mediates cell fate decisions during development and in adult tissues [131]. Upon ligand binding, Notch receptors undergo two successive proteolytic cleavages: an ectodomain cleavage followed by intramembrane proteolysis mediated by *γ*-secretase. This process releases the Notch intracellular domain (NICD), which translocates to the nucleus and binds CSL (an acronym for CBF-1/RBPJ-*κ* in Homo sapiens/Mus musculus respectively, Suppressor of Hairless in *Drosophila melanogaster*, Lag-1 in *Caenorhabditis elegans*) to activate its target genes like Hes-1 and Hes-5 which contain CSL binding sites [131–133]. The control of Notch receptors proteolysis, throughout the gastrointestinal tract, also regulates the intestinal injury/regenerative responses and drives intestinal inflammation and colon cancer initiation [129, 134].

The involvement of Wnt dysregulation in colorectal tumorigenesis is supported by its critical role in the differentiation of stem cells in intestinal crypts and in the maintenance of intestinal homeostasis [135]. Wnt signal transduction is based on the autocrine and paracrine interaction of secreted Wnt glycoproteins, rich in cysteine, essential for intestinal morphogenesis and for the maintenance of architecture and homeostasis in the adult intestinal epithelium [120, 136].

Furthermore, these pathways are necessary for the function of immune cells [137] of both innate and adaptive responses; in particular, they are necessary for the development of T lymphocytes [138]. For these reasons, it is not at all abstruse that the Wnt molecules seem to be also involved in immune diseases such as thyroiditis and psoriasis [139].

The aberrant regulation of Wnt/*β*-catenin signaling in the pathogenesis of colorectal cancer progression has long been recognized [140, 141], and it has been observed in 100% of CRCs [142]. Wnt proteins are about 40 kDa in size and contain many conserved cysteines [143]. They exhibit lipid modifications necessary for more efficient signaling and for both their secretion and ability to bind to Frizzled receptors. The changes by palmitoleic acid, a monounsaturated fat, linked to conserved serines seem to play an important role in the lipids for signaling [120]. The role of the lipids is also reflected by the requirement for Porcupine (Porc), a multipass transmembrane O-acyltransferase in the endoplasmic reticulum (ER), which is essential for Wnt palmitoylation and maturation, and is active only in Wnt-producing cells [120].

Importantly, the Wnt pathways transduce signals into many signaling cascades and protein phosphorylation amplifies the signal by modifying multiple substrate molecules. The signaling is activated by binding a Wnt protein ligand to a receptor of the Frizzled family, which sends the biological signal to the Dishevelled protein inside the cell. Three Wnt signaling pathways have been characterized: the *β*-catenin/canonical pathway, the noncanonical planar cell polarity (PCP) pathway, and the noncanonical Wnt/Ca2+ pathway. The noncanonical Wnt pathways control the activity of small GTPases altering the mechanism of the Actin and the cytoskeletal rearrangement; in this way, the Wnt molecules can stimulate JNK activity (N-terminal kinase c-Jun) or promote adhesion and cellular movement by calcium activating CaMK II and calmodulin [144]. In the absence of Wnt ligands, the cytoplasmic destructive complex of Adenomatous Polyposis Coli/Axin (*APC*/Axin) regulates the exit of the canonical Wnt pathway by controlling the stability of *β*-catenin in the cytoplasm, where it binds and phosphorylates the *β*-catenin through two constitutively active serine-threonine kinases (CK1a and GSK3a/b). This continuous elimination of *β*-catenin prevents it from reaching the nucleus and the Wnt target genes are then repressed by the DNA-bound T-cell factor/lymphoid enhancer factor (TCF/LEF) proteins [120]. The mutations in the molecules that are part of the Wnt/*β*-catenin pathway (in particular, the truncating mutations in the *APC* gene) lead to the formation of constitutive nuclear TCF/*β*-catenin complexes and to the uncontrolled transcription of target genes. This decompensation of Wnt signaling is present in at least 80% of colorectal carcinomas [145]. The *APC* protein, associated with the microtubule cytoskeleton, has an important effect on the structure and differentiation of intestinal epithelial cells. Thus, *APC* loss of functions in intestinal cells can lead to the polyp development [146, 147]. Recently, a novel role for *APC* mutated in reducing the action of the immune system has been suggested, preventing the control of intestinal inflammation. In particular, a deficiency in the *APC* protein reduces nuclear transcription factor NFAT, thereby preventing T-reg lymphocyte activation and then a failure in the control of local inflammation in the intestine [9]. Recent studies have shown that chronic inflammation and ROS production can activate the Wnt/*β*-catenin pathways, but the mechanisms involved remain unclear.

### 4.2. Oxidative Stress

Oxidative damage has been suggested to promote tumor initiation and progression by increasing mutation rates and activating oncogenic pathways [148]. On the other hand, also proinflammatory cytokines trigger oxidative stress that increases mucosal permeability and compromise the regenerative potential of the intestinal epithelium [149].

The most studied source of oxidative stress is attributable to the ROS formation. In fact, the intrinsic and extrinsic environmental stress factors, such as bacterial toxins or lipid overload, can induce ROS production through the activation of phagocytes and resident cells. This process involved the ROS production through microsomes, peroxisomes, and impairment of mitochondrial metabolism-related pathways [150, 151]. For some time, it has been proposed that inflammation is implicated in the onset of chronic degenerative diseases and tumors. This can be explained, at least in part, by overproduction and/or lack of H_2_O_2_ degradation.

Mitochondrial processes play an important role in tumor initiation and progression through alteration in glucose metabolism, production of ROS, and compromise of intrinsic apoptotic function [152]. Genetic and epigenetic alterations of Krebs cycle enzymes favour the shift of cancer cells from oxidative phosphorylation to anaerobic glycolysis. The process of maintaining redox homeostasis is driven by genome-wide transcriptional clustering with mitochondrial retrograde signaling and coupled with the glucose metabolic pathway and cell division cycle. Abnormalities of Krebs cycle enzymes cause ectopic production of Krebs cycle intermediates (oncometabolites) such as 2-hydroxyglutarate and citrate. These oncometabolites, which are important driving forces of cancer pathogenesis and progression, can stabilize hypoxia-inducible factor 1 (HIF1) and nuclear factor-like 2 (Nrf2), inhibit p53 and prolyl hydroxylase 3 (PDH3) activities, and regulate DNA/histone methylation, which in turn activate cell growth signaling. They also stimulate increased glutaminolysis, glycolysis, and production of ROS. Genetic alterations in Krebs cycle enzymes are also involved in increased fatty acid *β*-oxidations and induction of epithelial-mesenchymal transition (EMT) [153]. The central route for oxidative metabolism is the tricarboxylic acid (TCA) cycle responsible for the production of NADH and FADH2, which fuel the mitochondrial electron transport chain to generate ATP and source of metabolic intermediates required for anabolic reactions important for proliferating cells, which require the precursors for the synthesis of lipids, proteins, and nucleic acids [154].

The average of the ATP concentration over the cell cycle is higher and the pHi is globally more acidic in normal proliferating cells. The NAD+/NADH and NADP+/NADPH redox ratios are, respectively, five and ten times higher in cancer cells compared to the normal cell population as the effect of aerobic glycolysis or Warburg effect in cancer cells [155]. In normal proliferating cells, the Warburg effect is an example of homeostasis of redox status by transiently shifting metabolic flux from OXPHOS to glycolysis to avoid ROS generation during DNA synthesis and protect genome integrity [128]. In contrast, in tumor cells, the Warburg effect determines an alteration of the redox state derived from the glucose metabolic path reprogrammed by the OXPHOS dysfunction; this supports glycolysis and the excessive loss of ROS responsible for cancer progression [128]. For these reasons, the Warburg effect should be downregulated in the precancerous phase, while it should be used in the antitumor response since the response to oxidative stress can improve the action of targeted molecular agents. This implies that the antitumor cellular response induced by oxidative stress in the postcancerous phase should not be downregulated when cancer cells are still present [156].

Inflammation is associated with the production of ROS and oxidative damage of macromolecules such as 7,8-dihydro-8-oxoguanine (8-oxoG) in DNA. Most of these small base modifications are repaired by BER pathway by the pivotal role of the OGG1 and MUTYH glycosylases. OGG1 binds the 8-oxoG base with high affinity and then the complex interacts with canonical RAS family GTPases to catalyze the replacement of GDP with GTP, thus serving as a guanine nuclear exchange factor. OGG1-mediated activation of RAS leads to the phosphorylation of the mitogen-activated kinases MEK1,2/ERK1,2 and the increase of the downstream gene expression [10, 11]. A recent eminent study shows that OGG1 inhibition is able to alleviate inflammatory conditions in vivo [157]. MUTYH is a base excision repair glycosylase that removes adenine opposite 8-oxoguanine [158]. MUTYH has evolved from an OG:A mispair glycosylase to a multifunctional scaffold for rapid DNA damage response to a wide variety of DNA damaging signaling including PARP activation, ATR signaling, and SIRT6 activity. MUTYH inhibits the repair of alkyl-DNA damage and cyclopyrimidine dimers interaction with mismatch repair [159]. Many of the MAP variants encompass amino acid changes that occur at positions surrounding the two-metal cofactor-binding sites of MUTYH. One of these cofactors, found in nearly all MUTYH orthologs, is a [4Fe-4S]2+ cluster coordinated by four Cys residues located in the N-terminal catalytic domain [159] that may be a redox-sensitive target [160]. In the model of ulcerative colitis, Mutyh plays a major role in maintaining intestinal integrity by affecting the inflammatory response. Adenomas from Mutyh-/- mice had a greater infiltrate of Foxp3+ T regulatory cells, granulocytes, macrophages, myeloid-derived suppressor cells, and strong expression of TGF-*β*-latency-associated peptide and IL6. Then, MUTYH loss is associated with an increase in CRC risk involving immunosuppression and altered inflammatory response [161].

## 5. Major Colorectal Adenomatous Polyposis Syndromes

The major colorectal adenomatous polyposis syndromes that predispose to the development of CRC are divided into two groups based on predisposition: autosomal recessive and autosomal dominant disorders. The known autosomal recessive adenomatous polyposis syndromes are *MUTYH*-associated polyposis (MAP) and *NTHL1* associated tumor syndrome. *MUTYH* and *NTHL1* are DNA glycosylase genes of the BER. The first has a key role in the repair of oxidative DNA damage; the second, together with *OGG1*, does not contribute significantly to autosomal recessive polyposis [162]. BER is a single-strand DNA repair mechanism used by cells to maintain genomic integrity. This pathway is involved in the correction of events caused by oxidative damage, alkylation and deamination, and defects in its components give high-penetrant predisposition to develop polyposis [162]. For this reason, in these forms of polyposis, the greatest damage caused by oxidative stress occurs.

The MAP is the second most common high-penetrant Mendelian cancer syndrome associated with adenomatous polyposis [163]. Tumor sequencing has identified a specific mutation signature associated with germline *MUTYH* mutations (base excision repair defects), evidenced by an increase of somatic G:C to T:A base pair transversion [164]. This somatic signature might aid the invariant classification of rare *MUTYH* variants. Two variants c.536A>G, p.Tyr179Cys and c.1187G>A, p.Gly396Asp account for 70% to 80% of pathogenic *MUTYH* mutations in Europeans; homozygous p.Tyr179Cys mutations have a more aggressive phenotype than homozygous p.Gly396Asp or compound heterozygous p.Gly396Asp/p.Tyr179-Cys mutations [164, 165].

In the *NTHL1-*associated tumor syndrome, an association between base excision repair defects and specific somatic mutation signature in adenomas is found [162]. *NTHL1*, a novel recessive polyposis and CRC-predisposing gene, is the second DNA glycosylase gene of the BER pathway with a high-penetrant predisposition to develop polyposis. At first, p.Gln90 nonsense mutation was detected in the *NTHL1* gene, and then the other eight different pathogenic variants, all of which are nonsense or frameshift mutations, have been detected in patients with *NTHL1*-associated polyposis [162, 166, 167]. Patients develop multiple adenomatous polyps and CRC between the ages of 40 and 65 years, with a strongly resembling MAP phenotype [162, 166]. There are differences in the somatic mutation spectrum associated with the inactivation of the two DNA glycosylases. For *NTHL1*, somatic nonsense mutations involving C:G>*T*:A transitions were detected. The reason for this difference lies in the substrate specificity of the two enzymes. In the BER pathway, the 8-oxoguanine produced after oxidative damage is recognized and excised by the DNA glycosylase OGG1; then MUTYH removes the adenine base incorporated opposite to 8-oxoguanine [168]. Conversely, NTHL1 specifically targets oxidized pyrimidines and products of cytosine oxidation, which are strongly mutagenic due to their ability to mispair with adenine, causing C>T transitions [168]. Phenotypic characterization of additional families will increase the knowledge of tumor spectrum and cancer risk in association with *NTHL1-*associated tumor syndrome. Mutations in the *NTHL1* gene are extremely rare in the population and this syndrome is at least fivefold less frequent than MAP [167].

The polyposis-associated syndromes with autosomal dominant predisposition are familial adenomatous polyposis (FAP) and polymerase proofreading-associated polyposis (PPAP).

FAP accounts for approximately 1% of CRC and is the most common high-penetrant Mendelian syndrome that predisposes to adenomatous polyposis [169]. FAP is the major hereditary predisposition event leading to CRC development and is caused by truncating mutations in the *APC* gene [140, 170]. *APC* is essential for the development and homeostasis and its inactivation facilitates tumorigenesis. Heterozygotes develop multiple colonic polyps due to the loss of heterozygosity that for unclear reasons favours the growth of colonocytes in humans. Virtually, all patients with FAP will develop colorectal cancer unless the colon is removed. Somatic truncation mutations are also found in more than 80% of sporadic colorectal cancers and loss of heterozygosity (LOH) of chromosome 5q is found in 30–40% of CRC cases [171, 172]. Oncogenic *APC* mutations cluster in the mutation cluster region (MCR) [173]. *APC* is best known as a scaffold protein in the *β*-catenin destruction complex in the Wnt pathway, and its activity is antagonized by canonical Wnt signaling. Mutations in *APC* disrupt the degradation complex, deleting the Axin interaction domain that confers the turnover of *β*-catenin. This causes the stabilization of *β*-catenin and constitutive activation of the canonical Wnt pathway. Also, the loss of *APC* leads to the accumulation of nuclear *β*-catenin which activates the targets of the canonical Wnt pathway, the transcription factors T-cell factor (TCF), and lymphoid enhancer factor (LEF) [174]. *APC* alterations are an initiating event for sporadic CRC except for those carrying a CpG island methylator phenotype (CIMP) or hypermutable microsatellite instability (MSI) due to a defect in the MMR genes [175]. Patients with a familial risk of FAP have shown lower levels of ROS in the whole blood than patients with sporadic CRC [176]. This suggests that oxidative stress may play a crucial role in sporadic CRCs while its action could be less pivotal when the *APC* gene is mutated and CRC has an earlier onset. *APC* contributes to adenoma formation but some of its roles remain to elucidate. Inactivation of *APC* contributes to cancer development through processes besides the Wnt signaling. Conversely, recent evidences support the hypothesis of a new potential role of gain of function of *APC* truncations in colon cancer initiation and progression in addition to the loss of function [12]. Intracellular and/or oxidative microenvironment could promote the production of *APC* protein. This hypothesis is supported by the demonstration, in a recent paper, that in HT29 CRC cell line full-length *APC* requires mitochondrial respiratory ROS production to stimulate apoptosis [177]. But to better understand this paradox, first, it is necessary to examine the three models regarding *APC*. The two-hit hypothesis states that one of the copies of the gene is inactivated by a truncated mutation and the other by similar mutation or LOH [178]. The “three-hit hypothesis” was subsequently proposed, which states that mutant *APC* proteins retain some functions; thus, the third hit could affect the residual part of the gene with copy number gains or deletions [179]. The hypothesis of the functional implications of *β*-catenin shuttling is poorly understood. It might be possible according to the theory called “just-right nuclear export activity” that the loss of the central nuclear export signals—*adjacent to the MCR region*—has reduced nuclear export activity that compromises *APC* tumor-suppressing function [180, 181].

A novel function of *APC* is also regulating DNA repair modulating BER [182]. *APC* has also been found to shuttle into the nucleus, where it inhibits the assembly of base excision repair [183, 184] directly binding to APE1 endonuclease. The DNA repair inhibitory (DRI) domain of *APC* is located in the N-terminal region and is retained in *APC* mutants, allowing CRC cells to accumulate genetic alterations and to be more susceptible to DNA damaging chemotherapeutic agents [12].

**Figure 1:**
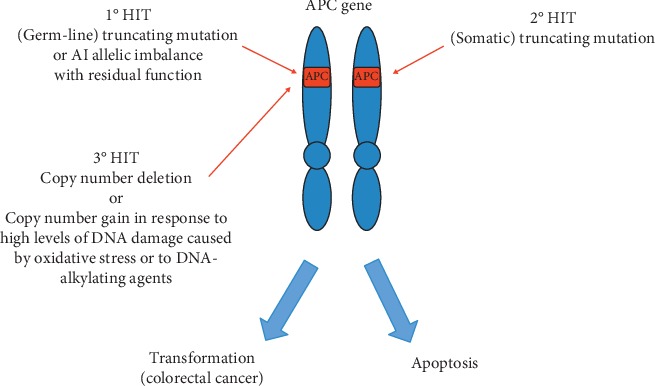
Hypothetical model of interaction between APC and oxidative stress in carcinogenesis. The figure shows a hypothetical model of inactivation of the APC gene that takes into account the influence of oxidative stress. In this model, the known “three-hit hypothesis” is further updated stating that mutant APC protein retains some functions. Thus, the third hit could affect the residual part of the gene with copy number gains or deletions. We now propose APC copy number gain in response to high levels of DNA damage caused by oxidative stress or by DNA-alkylating agents.

In this context, the *APC* role in apoptosis supports tumor suppressor function. In those cells with a high extent of damaged DNA, the *APC* level increases and blocks BER leading to apoptosis. But the overall role of *APC* in carcinogenesis remains paradoxal [182]. Thus, this function of *APC* to unbalance BER may favour chromosome instability (CIN) and then carcinogenesis (Figure 1). In this scenario, since *APC* does not help the repair of DNA damage, for example, from chemotherapeutic agents that are therefore more effective in a CRC cell with an *APC* mutation than with wild type *APC*, indeed with increased *APC* levels, the CRC cell, thus damaged, undergoes apoptosis.

Another mechanism of inactivation is a decrease in transcript expression, as previously described [185, 186], in which *APC* showed a reduced germline expression regardless of the presence of the mutation.

Of the ∼20% of sporadic CRCs that have intact *APC* gene, many contain mutations in the N-terminal phosphorylation sites in *β*-catenin, sites that mediate its proteosomal degradation. This could represent a compelling genetic argument that *APC* suppresses intestinal neoplasia through the inhibition of the canonical Wnt/*β*-catenin pathway.

PPAP is an autosomal dominant syndrome caused by monoallelic germline mutations in the exonuclease (proofreading) domains of *POLE* and *POLD1* [187–189]. *POLE* and *POLD1* encode ε and δ polymerases, respectively, and accurate proofreading via their exonuclease domains is required to correct mispaired bases inserted during DNA replication*. POLD1* (*Polδ*) has exonuclease and polymerase activities and is critical in BER and MMR [190]. Both *POLE* and *POLD1* have been associated with an increased risk of endometrial cancer and furthermore *POLD1* has been associated with breast and brain tumors in addition to CRC and endometrial cancer [191]. One of the common germline variants in *POLE*, encoding the pathogenic p.Lys424Val, has been encountered with variable frequencies in different cohorts of individuals with unexplained polyposis and/or early-onset and familial CRC [190, 191]. In PPAP patients, the proofreading activity of *POLE* or *POLD1* is impaired, whereas the polymerase activity is unaffected. As a consequence, these patients accumulate base substitutions during life, which eventually results in the development of hypermutated tumors [190, 191]. The clinical phenotype of PPAP has not yet been precisely established, but the data strongly indicate that PPAP results in a high-penetrant predisposition to develop polyposis, early-onset CRC, and extracolonic tumors, including endometrial, stomach, and duodenal tumors [190, 191].

The study from Haraldsdottir supported the hypothesis of the coexistence of somatic MMR alterations with somatic mutations in DNA polymerase *POLE* or *POLD1* in patients with hypermutated colon and endometrial cancers without germline MMR mutations [192]. It is not yet clear if mutations in the DNA polymerase genes are the initiating events causing MMR gene mutations or vice versa. As the critical role of *POLD1* in BER as in MMR, the potential function of oxidative stress in PPAP polyposis should be investigated.

Serrated Polyposis Syndrome (SPS) is another genetic disease of the colon whose inheritance remains unknown: both autosomal recessive and autosomal dominant patterns have been suggested [193]. The serrated lesions, unlike conventional adenomas which are uniformly dysplastic, contain no dysplasia and include the hyperplastic polyps. Approximately half of the cancers in the serrated pathway have microsatellite instability. Sessile serrated polyps, common in the proximal colon, the same location where hypermethylated cancers are more common, have a high prevalence of mutations in the *BRAF* oncogene and hypermethylation due to epigenetic inactivation of the promoter region of *MLH1* gene. Some evidences suggest that the sessile serrated polyp-to-cancer sequence takes 10 to 20 years, the same time frame generally accepted for the conventional adenoma-to-cancer sequence. The serrated neoplasia is characterized by the presence of multiple serrated adenomas including sessile and traditional serrated adenomas [194]. First- and second-degree relatives of individuals with SPS are at increased risk of developing CRC, which occurs on average in subjects aged between 50 and 60 years, but the percentage remains uncertain [195]. The serrated pathway is also characterized by mutations of *KRAS* which, together with *BRAF*, are thought to initiate the development of serrated adenomas via activation of the MAP kinase pathway. Activation of Wnt signaling in the progression of this kind of adenoma to carcinoma is less clear and in the past individuals with SPS were tested for *APC* and *MUTYH* mutations, founding some missense *APC* mutations in patients with serrated pathway neoplasia. Other affected individuals were found to carry a mutation in *SMAD4, BMPR1A,* and *PTEN* [196–198]. To date, the genetic basis of SPS remains to be determined. Recently, a germline mutation in RING finger protein 43 (*RNF43*) was found to segregate in a family with SPS phenotype [199]. *RNF43* encodes an E3 ubiquitin ligase that negatively regulates Wnt signaling [200]. Somatic *RNF43* mutations have been identified in up to 18% of CRCs with molecular features of SPS [201, 202].

## 6. Conclusion and Therapeutic Implications

The molecular biology of early carcinogenesis is controlled by genomic susceptibility, metabolic reprogramming, and microenvironment.

The development of adenomatous polyps passes through the interaction between oxidative stress and pathways involved in the colon epithelium renewal and immune defense. Recent studies have shown that chronic inflammation and oxidative stress can activate the Wnt/*β*-catenin pathways, but the mechanisms involved remain unclear. The most studied source of oxidative stress is ROS whose production is induced through the activation of phagocytes and resident cells after stimulation by environmental stress factors, such as bacterial toxins or lipid overload. Mitochondria may play an important role in tumor initiation and progression through metabolic processes and ROS production.

Inflammation is also associated with oxidative damage of macromolecules such as 7,8-dihydro-8-oxoguanine (8-oxoG) in DNA. Most of these small base modifications are repaired by the BER. The administration of NSAIDs may contribute to the downregulation of the canonical Wnt/*β*-catenin pathways acting as PPAR agonists. In fact, PPAR agonists can reduce inflammation, oxidative stress, and proliferation promoting cell cycle arrest, cell differentiation, and apoptosis.

As regards ROS, data on their role in the pathogenesis of colorectal cancer are accumulating. Patients with advanced cancer of the colon, pancreas, and breast showed extensive granulocyte activation with the release of ROS, which could be an important factor in the process of carcinogenesis.

In the control of intestinal inflammation, a new role for mutated *APC* has been suggested in reducing the action of the immune system. In particular, a deficiency in the *APC* protein reduces nuclear transcription factor NFAT, thereby preventing T-reg lymphocyte activation and then a failure in the control of local inflammation in the intestine.

Given the multiple roles of *APC*, new therapeutic opportunities could be addressed. *APC* restoration leading to tumor regression has been observed. In this model, the reacquisition of the self-renewal and multilineage differentiation capability and reestablishment of the normal crypt-villus homeostasis can restore homeostasis in the intestinal crypt. Moreover, the function of *APC* protein in regulating DNA repair is very important as a therapeutic implication making DNA damaging chemotherapeutic agents more effective in CRC cells that tend to accumulate mutations. Additional studies will determine whether approaches based on Wnt inhibition would provide long-term therapeutic value in CRC, but it is clear that *APC* disruption plays a central role in driving and maintaining tumorigenesis.

The findings that high levels of ROS were found in the blood of patients with sporadic CRC and in the normal-appearing rectal mucosa of patients with history of CRC, compared to patients with familial risk of FAP, not only confirm the crucial role of oxidative stress in CRC but also suggest a minor role of ROS when *APC* expression is completely lost, as in patients with FAP.

Also, microbiota may influence microenvironmental homeostasis and differential expression of Wnt signaling components. The association between loss-of-function mutations in genes of different pathways, including Wnt, and shifts in the abundances of specific sets of bacterial taxa are suggestive of potential functional interactions. Differences in lifestyles and exposition to environmental factors may induce microbiota changes and immune response modulation in the colon epithelial microenvironment on the light of homeostatic adaptation to the oxidative stress, thus inducing adenoma onset in specific colorectal areas. The therapeutic approach should take into account the composition of the microbiota and the levels of oxidative stress.

So in this review ultimately we conclude that oxidative stress may be important in inducing colorectal adenomas through the interaction with Wnt signaling and DNA damage response. A deeper insight into the pathophysiological mechanisms influencing the crosstalk among Wnt signaling and/or microbiota dysregulation, oxidative stress, and DNA damage could contribute to better elucidate the causes responsible for CRC onset, with the aim of providing new clinical approaches for the prevention and develop innovative therapeutic strategies for this tumor.

## Figures and Tables

**Table 1:** Possible risk factors associated with colorectal adenoma onset.

Risk factors	Effects on adenoma onset	References
Age	Possible onset before 50 years	[26, 27]
	Major incidence after 50 years	[28]
	Peak at 70 years	[16]

Gender	Higher incidence in males than in females	[16, 29]

Ethnicity	High incidence in Western and African Americans populations	[4, 30, 31]

Persistent organic pollutants		
Organochlorine pesticides	Wnt and Hedgehog/Gli1 pathway activation. Increased ROS	[32]
*Dichlorodiphenyldichloroethylene*		
Polychlorinated biphenyls	Predisposition to gut inflammation	[33]
*Dioxin-like PBS126*		

Heavy metals		
*Arsenic*	Altered gut-associated immunity and microbiome	[34]
*Cadmium*	Intestinal inflammation, modified microbiome	[35]

Antibiotics	Changes in gut microbiota	[36]

Food additives	Changes in gut microbiota	[36]

Diet		
*Excessive processed red meat consumption*	Gut dysbiosis by HCA and PAH production. Heme iron associated with aldehyde generation	[37–40]
*High saturated fat intake*	Intestinal inflammation	[41–43]

Lifestyles		
*Stress*	Increased stress hormones, altered rate of cell growth	[44, 45]
*Lack of physical exercise*	Overweight and obesity	[46]

Obesity	High TNF-*α*, IGF-1, and adiponectin	[47]

Cigarette smoking	Oxidative stress, chronic inflammation, genetic/epigenetic alterations by BaP/HCA generation	[25, 48–50]

Heavy alcohol drinking	Production of acetaldehyde	[51]

Gut microbiota alterations		
*Fusobacterium nucleatum*	Colon cells adhesion by FadA, *β*-catenin activation	[52]
*Escherichia coli*	Inflammation and DNA breaks by CDT and colibactin	[51]
*Bacteroides fragilis*	Wnt pathway activation by BFT. ROS production	[53]
*Enterococcus faecalis*	Superoxide production	[54]
*Helicobacter pylori*	Increased serrated polyps	[55]

Bacteroides fragilis toxin (BFT); cytolethal distending toxin (CDT); heterocyclic amines (HCAs); polycyclic aromatic hydrocarbons (PAHs); benzo[a]pyrene (BaP).

## Abbreviations

8-oxoG: 7,8-dihydro-8-oxoguanine
ADAM: A disintegrin and metalloproteinases
*APC*: Adenomatous polyposis Coli
APE: Apurinic/Apyrimidinic *Endonuclease*
ATR: Serine/threonine-protein kinase
BER: Base excision repair
BMI: Body mass index
BMP: Bone morphogenetic protein
BMPR1A: Bone morphogenetic protein receptor, type IA
*BRAF*: B-Raf proto-oncogene, serine/threonine kinase
CIN: Chromosome instability
CIMP: CpG island methylator phenotype
COX-2: Cyclooxygenase 2
CaMK II: Ca ^2+^ /calmodulin-dependent protein kinase II
CRC: Colorectal cancer
DNMT: DNA methyltransferases
ERK: Extracellular signal-regulated kinase
FAD/FADH: Flavin adenine dinucleotide/semiquinone
FPR: Formyl peptide receptors
GSK3: *Glycogen synthase kinase 3*
Hg: Hedgehog
HNE: 4-hydroxynonenal
HIF: Hypoxia-induced factor
IGF-1: Insulin-like growth factor
IFNs: Type I interferons
IL-1R: Interleukin-1 receptor
IL-6: Interleukin 6
JAK: Janus kinase
JNK: c-Jun N-terminal kinase
LDL: Low-density protein
MAP: MUTYH-associated polyposis
MAPK: Mitogen-activated protein kinase
MIBE: Microbiome-induced bystander effects
MHL1: MutL homolog 1
MMR: DNA mismatch repair
NADPH: Nicotinamide adenine dinucleotide phosphate
NAFLD: Nonalcoholic fatty liver disease
NER: Nucleotide excision repair
NFAT: Nuclear factor of activated T-cells
NICD: Notch intracellular domain
NSAIDs: Nonsteroidal anti-inflammatory drugs
NF-kB: Nuclear factor-kB
NOX: NADPH oxidase
NOXO1: NADPH oxidase organizer 1
NRF2: Nuclear factor erythroid 2-related factor 2
NTHL1: Nth like DNA glycosylase 1
KRAS: Kirsten RAt Sarcoma
OCPs: Organochlorine pesticides
OGG1: 8-oxoguanine DNA glycosylase-1
OXPHOS: Oxidative phosphorylation
PPAP: Polymerase proofreading-associated polyposis
PARP: Poly (ADP-ribose) polymerase
PDH3: Prolyl hydroxylase 3
PI3K: Phosphoinositide-3-kinase–protein kinase
PTEN: Phosphatidylinositol 3,4,5-trisphosphate 3-phosphatase and dual-specificity protein phosphatase
PCBs: Polychlorinated biphenyls
*POLD1*: Polymerase delta 1
*POLE*: DNA polymerase epsilon catalytic subunit A
PPAR: Peroxisome proliferator-activated receptors
ROS: Reactive oxygen species
RFP43: Ring Finger Protein 43
SCFAs: Short-chain fatty acids
SIRT6: Sirtuin 6
SMAD: Small mother against decapentaplegic
STAT: Signal transducers and activators of transcription
TCA: Tricarboxylic acid
TAZ: Transcriptional coactivator with PDZ-binding motif
TILs: Tumor-infiltrating lymphocytes
TNF: Tumor necrosis factor
TCF/LEF: T-cell factor/lymphoid enhancer factor
TGF-*β*: Transforming growth factor-beta
TLR: Toll-like receptors
WNT: Wingless/It
YAP: Yes-associated protein.
